# Successful cryopreservation of coral larvae using vitrification and laser warming

**DOI:** 10.1038/s41598-018-34035-0

**Published:** 2018-10-24

**Authors:** Jonathan Daly, Nikolas Zuchowicz, C. Isabel Nuñez Lendo, Kanav Khosla, Claire Lager, E. Michael Henley, John Bischof, F. W. Kleinhans, Chiahsin Lin, Esther C. Peters, Mary Hagedorn

**Affiliations:** 1 Smithsonian Conservation Biology Institute, Front Royal, VA 22630 United States of America; 2 Hawaii Institute of Marine Biology, 46-007 Lilipuna Rd, Kaneohe, HI 96744 United States of America; 3 Department of Mechanical Engineering, University of Minnesota at Twin Cities, 111 Church St SE, Minneapolis, MN 55455 United States of America; 4 Department of Biomedical Engineering, University of Minnesota at Twin Cities, 312 Church St SE, Minneapolis, MN 55455 United States of America; 5 Department of Physics, Indiana University-Purdue University Indianapolis, Indianapolis, IN 46202 United States of America; 6 National Museum of Marine Biology & Aquarium, Pingtung, 944 Taiwan; 7 Institute of Marine Biology, National Dong Hwa University, Pingtung, 944 Taiwan; 8 Environmental Science and Policy, George Mason University, Fairfax, VA 22010 United States of America

## Abstract

Climate change has increased the incidence of coral bleaching events, resulting in the loss of ecosystem function and biodiversity on reefs around the world. As reef degradation accelerates, the need for innovative restoration tools has become acute. Despite past successes with ultra-low temperature storage of coral sperm to conserve genetic diversity, cryopreservation of larvae has remained elusive due to their large volume, membrane complexity, and sensitivity to chilling injury. Here we show for the first time that coral larvae can survive cryopreservation and resume swimming after warming. Vitrification in a 3.5 M cryoprotectant solution (10% v/v propylene glycol, 5% v/v dimethyl sulfoxide, and 1 M trehalose in phosphate buffered saline) followed by warming at a rate of approximately 4,500,000 °C/min with an infrared laser resulted in up to 43% survival of *Fungia scutaria* larvae on day 2 post-fertilization. Surviving larvae swam and continued to develop for at least 12 hours after laser-warming. This technology will enable biobanking of coral larvae to secure biodiversity, and, if managed in a high-throughput manner where millions of larvae in a species are frozen at one time, could become an invaluable research and conservation tool to help restore and diversify wild reef habitats.

## Introduction

Coral reefs are imperiled globally by ocean warming and acidification resulting from the overuse of fossil fuels^1^. Since the early 1980s, three global bleaching events and scores of regional bleaching events have caused extensive coral stress and mortality^2–4^. On the Great Barrier Reef in Australia, above-average sea surface temperatures during 2016 and 2017 caused back-to-back mass bleaching events for the first time, resulting in the loss of an estimated 29% of shallow water coral cover in 2016 alone^5^. Even when coral survive bleaching, reproduction and fecundity are often profoundly affected for multiple breeding seasons^6^, and the stress caused by warming can result in reduced reproductive output even in corals that do not visibly bleach^7^. Over three-quarters of the world’s coral reefs are predicted to experience annual bleaching by the end of this century^8^, so the need for innovative restoration tools to conserve and secure reef biodiversity is critical.

One of the most effective methods to secure biodiversity is low-temperature storage of reproductive material in biorepositories. Ultra-cold storage of living coral samples from healthy reefs can help mitigate the loss of genetic and species diversity caused by natural disasters and major bleaching events while avoiding problems like genetic drift that occur in multi-generational *ex situ* populations^9^. Storage of genetic material would also support and complement existing reef restoration programs, which encompass a range of strategies. These include research aimed at improving the ability of coral to adapt to changing environmental conditions (such as assisted gene flow, selective breeding, and hybridization)^10^ and improving restoration methods by diversifying depauperate populations with frozen sperm. To be effective, these restoration efforts will require a broad base of species and genetic diversity. Therefore, it is vital to begin globally-linked programs that can secure reef biodiversity over a broad swathe of populations while diverse reef populations still exist. The present study provides an important component for future reef restoration: successfully cryopreserved coral larvae.

Coral sperm have been been successfully cryopreserved for the past 6 years^11^ and these frozen assets have been transported thousands of kilometers to be used in *ex situ* fertilization experiments that produced new coral settlers^12^. However, the use of frozen sperm for reproduction requires access to fresh oocytes, which are only available for a few days each year. Cryopreservation of coral embryos or larvae would circumvent some of these challenges.

Cryopreservation of mammalian embryos has been possible for over thirty years, and today vitrification is used to cryopreserve embryos from a range of species including humans, mice, and cattle^13–15^. Despite this long history of success in some mammalian species, embryo cryopreservation is still not possible for the majority of non-mammalian species such as birds, reptiles, amphibians and fishes. The many challenges of non-mammalian embryos include their large size (>0.5 mm diameter), a large and fatty yolk, chill sensitivity, and barriers to permeability that impede the movement of water and cryoprotectants across cell and compartment membranes^16,17^. Recently, Khosla *et al*.^18^ reported the first successful cryopreservation of a non-mammalian embryo, the zebrafish (*Danio rerio*), using microinjected gold nanoparticles with vitrification and ultra-rapid laser warming^19,20^. Coral larvae share several physiological parameters with zebrafish embryos and present many of the same challenges to cryopreservation. Specifically, they are relatively large (around 200–600 µm diameter), lipid-rich, and chill sensitive^21^. With this in mind, we hypothesized that vitrification and laser warming would be a suitable approach to cryopreservation of coral larvae.

To examine vitrification and laser warming of coral larvae, we first determined the rates of water and cryoprotectant movement across coral larval cell membranes in *Fungia scutaria*. From this information, we designed two vitrification solution toxicity tests for *F. scutaria* larvae at different developmental stages. The selected cryoprotectant solution and developmental stages were used for vitrification and laser warming, resulting in the production of live, swimming coral larvae. This proof-of-concept experiment forms the framework for future experiments that will examine the applicability of vitrification and laser warming to larger coral larvae, such as the reef-building acroporids, and determine whether larvae that have been cryopreserved and warmed can find their symbionts and settle.

## Results

### Larval development of *Fungia scutaria*

**Figure 1:**
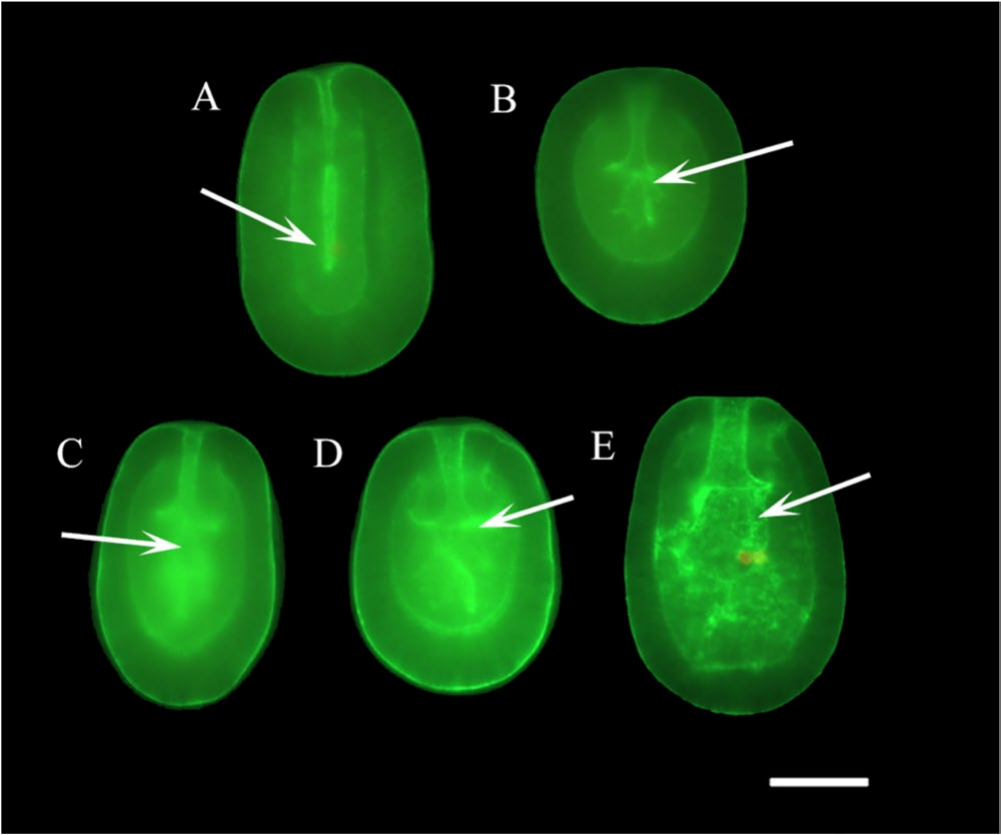
Phalloidin Staining: Wholemount phalloidin fluorescent microscopic images of developing *F. scutaria* larvae stained for actin with AlexaFluor 488-conjugated phalloidin. This stain highlights the growth and development of the gastrovascular cavity (GVC; white arrows) and internal structure. Bar = 40 µm. (**A**) Day 1, the GVC was elongated and flat. There was a clear delineation between the endoderm and ectoderm but it appeared to be incomplete in the apical region. The plasmalemmas of cells lining the developing gastrovascular cavity were brightly stained. (**B**) Day 2, the actinopharynx began to open and widen and the mesenteries began to develop in the GVC. Additionally, there was more complete development of the endoderm and mesoglea. (**C**) Day 3 showed a well-developed actinopharynx and mouth and more complex GVC. (**D**) Day 4, the mesoglea began to thicken and complexity of the GVC continued to increase. (**E**) Day 5 presented a continued widening of muscular actinopharynx and development of mesenteries.

Larval age was considered a potential issue for vitrification and laser warming because the increasing structural complexity would likely impede the movement of water and cryoprotectants and in doing so increase the chances of ice crystal formation. Towards that end, we used AlexaFluor 488-conjugated phalloidin to label actin to visualize the increase in structural complexity of the developing larvae. AlexaFluor 488-conjugated phalloidin labelling showed the growth and development of the mouth, actinopharynx, and gastrovascular cavity in larvae from Day 1 to Day 5 post-fertilization. On Day 1 the gastrovascular cavity was narrow and elongated, and over subsequent days the mouth and actinopharynx widened and the complexity of the larvae increased as tissue layers developed and mesenteries formed within the gastrovascular cavity (Fig. 1). By Day 4, larvae had a very complex gastrovascular cavity and by Day 5 the larvae were capable of infection with *Symbiodinium* (Fig. 1). Developmental changes to larval structure from Day 1 to Day 4 post-fertilization are described in greater histological detail in the Supplemental Information.

### Movement of water and cryoprotectants across larval membranes

Although previous work has addressed some aspects of cell membrane permeability of *F. scutaria* larvae to cryoprotectants^21,22^, we wanted to review these parameters during larval development with a more rigorous view toward the requirements of vitrification and laser warming. To this end, we examined larval volume changes in response to cryoprotectant exposure. As with previous studies^13,21^, we interpreted a reduction in larval volume in response to cryoprotectants as an indication of cell dehydration, and a subsequent increase in larval volume to indicate re-swelling of cells due to cryoprotectant permeation.

**Figure 2:**
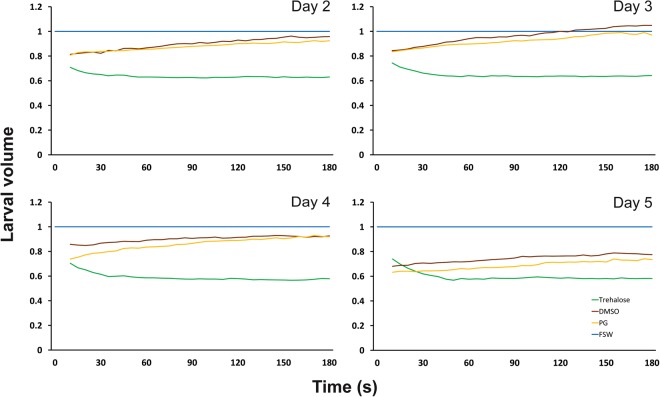
Changes in larval volume in response to the permeating cryoprotectants dimethyl sulfoxide (DMSO; 10% v/v in FSW; brown) and propylene glycol (PG; 10% v/v in FSW; yellow), and the non-permeating cryoprotectant trehalose (0.75 M in 50% FSW, green), compared to control larval volume in filtered seawater (blue), over a three-minute period. A reduction in larval volume was observed within 1 minute of exposure to permeating and non-permeating cryoprotectants on all four days. Increasing larval complexity affected the permeability of DMSO and PG from Day 2 to Day 5. Permeation of DMSO and PG caused larval volume to return to the control volume within the 3-minute analysis period on Days 2 and 3, but larval volume only returned to 85–90% of control volume on Day 4 and 75–80% on Day 5. Volumes are expressed as a proportion of the mean larval volume in FSW for a given day, and each data point is a mean of n ≥ 10 measurements.

Two- to five-day-old larvae exposed to 0.75 M trehalose in 50% filtered seawater (FSW) showed rapid dehydration, reducing in volume by 35–45% after one-minute exposure and remaining at this size for the remainder of the three-minute observation period (Fig. 2). Larvae exposed to dimethyl sulfoxide (DMSO) and propylene glycol (PG) in FSW showed a similar rapid reduction in volume, but this was followed by gradual re-swelling as cryoprotectants entered the cells to replace water lost during dehydration (Fig. 2). We observed an increase in the amount of dehydration in response to DMSO and PG exposure as the larvae developed from two to five days of age, but there was a concomitant reduction in cryoprotectant permeation over this same period. For example, in Day 2 larvae there was an initial reduction in volume of ~20% and almost all of this volume was replaced by cryoprotectant after three minutes’ exposure, whereas Day 5 larvae showed a 30–35% reduction in volume with minimal re-swelling (Fig. 2).

In addition to the volume changes, image sequences of larvae at ×100 magnification on a compound microscope showed changes to the appearance and structure of the larvae in response to cryoprotectant exposure that indicated that damage was occurring. Damage usually began with localized destablization of the body wall of the larvae after around 1.5 minutes’ exposure to cryoprotectants and eventually resulted in disintegration of larval tissues. In late Day 4 and Day 5 larvae this was often accompanied by expulsion of material from the actinopharynx.

### Larval rehydration and recovery following exposure to cryoprotectant solutions

We designed two vitrification solutions based on the cryoprotectants assessed in the first experiment. Solution 1 consisted of 7.5% v/v DMSO, 7.5% v/v PG, and 1 M trehalose in PBS, and solution 2 consisted of 5% v/v DMSO, 10% v/v PG, and 1 M trehalose in PBS. Both solutions contained approximately 3.5 M of solutes. The toxicity of these cryoprotectant solutions to *F. scutaria* larvae was determined by assessing the survival of larvae following a 1-minute exposure to cryoprotectant solution followed by rehydration.

**Figure 3:**
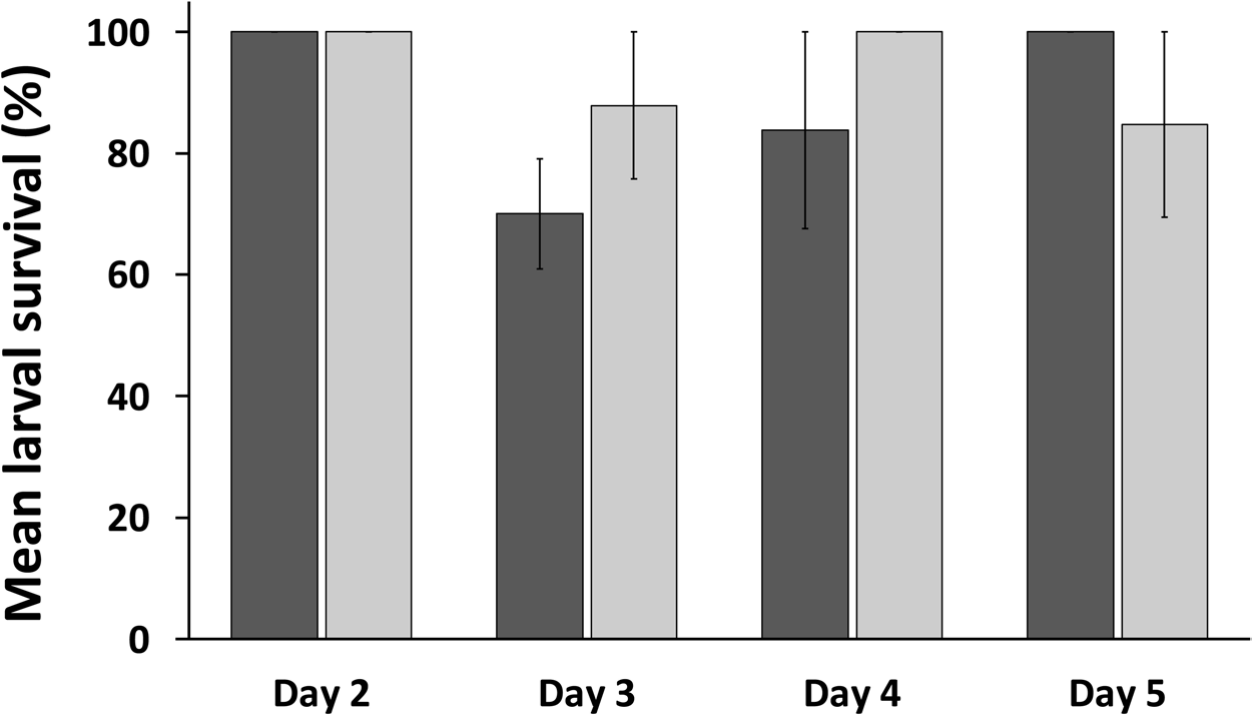
Toxicity experiments measure the proportion of larvae exposed to two different cryoprotectant solutions (cryoprotectant solution 1: 7.5% (v/v) DMSO, 7.5% (v/v) PG, and 1 M trehalose in PBS, dark columns; cryoprotectant solution 2: 5% (v/v) DMSO, 10% (v/v) PG, and 1 M trehalose in PBS, light columns) that resumed swimming at two hours post-rehydration. Solution 2 demonstrated a better than 80% survival on all developmental days, whereas Solution 1 was slightly more damaging to larvae, especially on Day 3. No significant differences were found among any of the cryoprotectant solutions or days (*P* > 0.05; Kruskal-Wallis test with Dunn’s multiple-comparisons test). Data are mean ± SEM of n = 3 technical replicates. Each replicate contained 20–40 larvae from a pooled sample of larvae from 10 females.

Recovery of larvae exposed to cryoprotectant solutions was observed on all four days, and the proportion of larvae that resumed swimming by two hours after rehydration varied with larval age. Day 2 larvae had the highest cryoprotectant tolerance, while recovery of larvae on Days 3, 4, and 5 varied. By two hours after rehydration, 100% of the Day 2 larvae exposed to either cryoprotectant solution had resumed swimming (Fig. 3) and the appearance of the larvae was not noticeably different from untreated control larvae. Larval tolerance to both cryoprotectant solutions decreased on Day 3, with only 70% of larvae exposed to solution 1 and 88% of larvae exposed to solution 2 resuming swimming. Recovery was improved somewhat in both solutions on Day 4, with 84% and 100% recovery of larvae exposed to solutions 1 and 2 respectively (Fig. 3). In contrast to Days 3 and 4 where larval recovery was higher following exposure to solution 2 compared to solution 1, on Day 5 100% of larvae exposed to solution 1 resumed swimming whereas 85% recovered after exposure to solution 2. Despite these apparent differences, there were no significant differences in recovery among any of the days or cryoprotectant solutions tested (*P* > 0.05).

### Vitrification and laser warming of *Fungia scutaria* larvae

It was decided to focus on Days 2 and Day 3 larvae for vitrification and laser warming experiments since the first experiment showed that larvae from these stages had less structural complexity and a greater level of dehydration than larvae from Days 4 and 5. Cryoprotectant solution 2 was chosen for vitrification and laser warming trials because of the higher rates of recovery of larvae from Days 2 and 3 following exposure and rehydration compared to solution 1, and because observations from the first experiment indicated that larval membranes were more sensitive to DMSO than to PG. A total of 523 larvae (283 on Day 2 and 240 on Day 3) were vitrified and laser-warmed. Larvae were vitrified in 1 µL droplets of cryoprotectant solution 2 containing gold nanorod particles on a modified cryotop, and warmed at a rate of approximately 4,500,000 °C/min with 10.9J of infrared laser energy. Rehydrated larvae were transferred into FSW in a 24-well plate (one cryotop per well) and larval survival was assessed the following day (approximately 12–18 hours post-rehydration) by counting the number of larvae that were swimming in each well.

**Figure 4:**
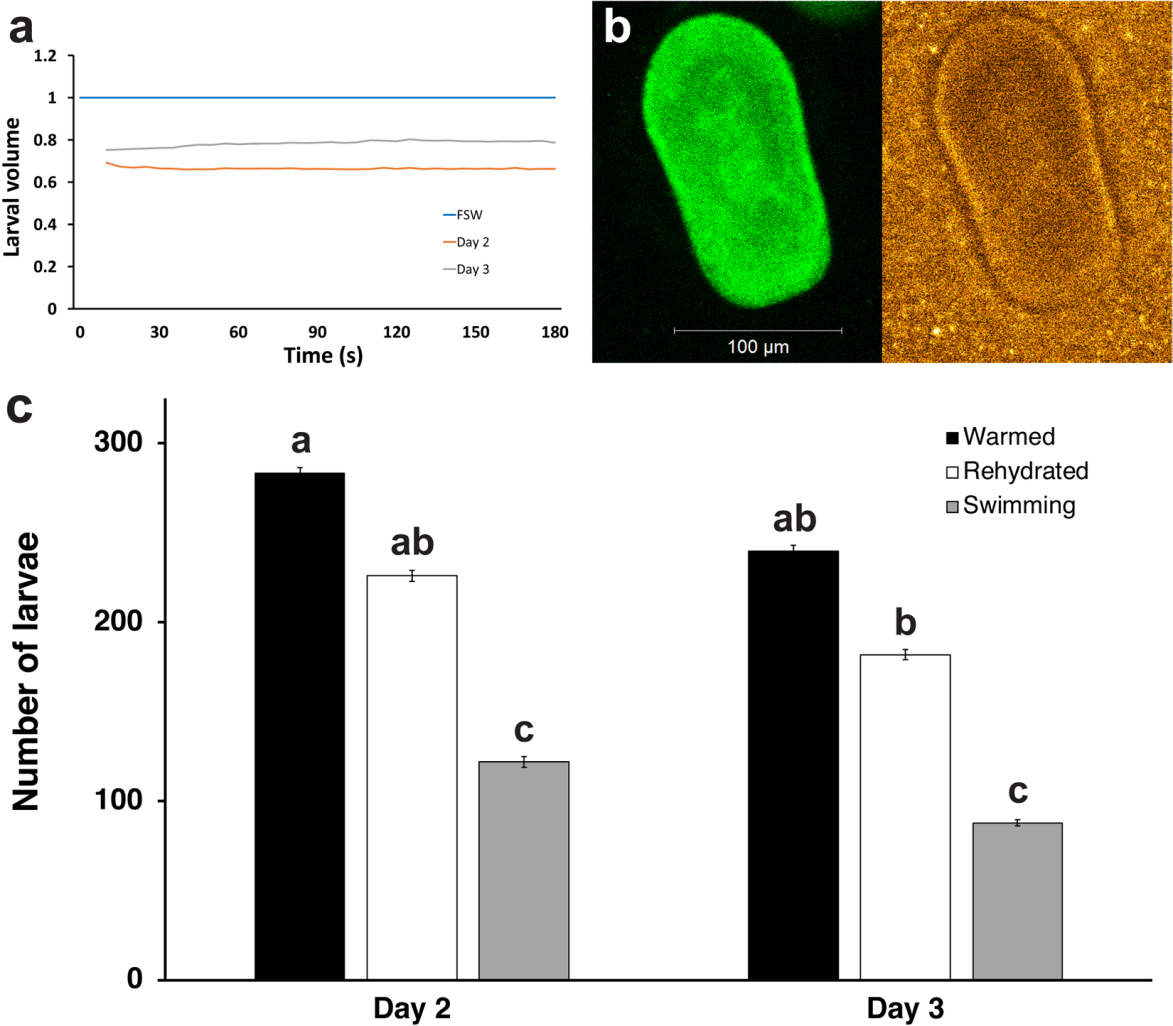
Dehydration of larvae in response to complex cryoprotectant solutions reduced internal ice crystal formation during vitrification and even distribution of the gold nanorod particles permitted reproducible melting of 1-µL vitrified droplets, resulting in live *F. scutaria* larvae post-warming. (**A**) Changes in larval volume in response to cryopreservation solution 2 (5% v/v DMSO, 10% v/v PG, and 1 M trehalose in PBS) in larvae from Day 2 and Day 3 post-fertilization. Dehydration was more effective on Day 2 than on Day 3, which may account for the higher rate of larval recovery on that day. Volumes are expressed as a proportion of the mean larval volume in FSW for a given day, and each data point is a mean of n ≥ 10 measurements. (**B**) Confocal images of a Day 3 larva showing fluorescence from GFP (left) and distribution of DyLight-coated gold nanorod particles surrounding the larva in cryoprotectant (right). Gold nanorod particles were evenly distributed in the cryoprotectant solution and formed a dense field that completely surrounded the larvae. (**C**) Total number of Day 2 and Day 3 larvae that were vitrified and laser warmed (black), compared to the number remaining after rehydration (white), and the number that resumed swimming (grey). The number of larvae that survived rehydration and resumed swimming was reduced on Day 3 compared to Day 2 but this was not significant. Data are mean ± SEM. Columns with the same letter are not significantly different (*P* > 0.05, Kruskal-Wallis test with Dunn’s multiple-comparisons test).

There was a difference in the amount of dehydration that occurred in response to the complex cryoprotectant solution on Day 2 compared to Day 3. On Day 2, larval volume was reduced by ~34% after 60 s exposure to cryoprotectant solution 2, while on Day 3 larval volume was only reduced by ~22% at the same time point. Larval volume did not change for the remainder of the three-minute exposure time on either day, indicating that minimal cryoprotectant permeation was occurring (Fig. 4A). Confocal imaging confirmed that gold nanorod particles suspended in cryoprotectant solution were evenly distributed around the larvae (Fig. 4B), which resulted in consistent melting of 1-µL vitrified sample droplets.

The number of larvae per sample droplet ranged from 8 to 20 (mean = 12.5 ± 0.5 larvae) and there was no observed effect of larval number on vitrification and laser warming within this range. There was no significant loss of larvae during warming and rehydration on either day (*P* > 0.05), with 80% (226 of 283 larvae) of Day 2 and 76% (182 of 240 larvae) of Day 3 larvae that were laser-warmed making it through the rehydration process (Fig. 4C). Likewise, the proportion of larvae that resumed swimming after vitrification and laser warming was higher for Day 2 larvae (43%—122 larvae) than for Day 3 larvae (37%—88 larvae) (Fig. 4C). In addition, the proportion of larvae that showed abnormal movement post-warming was higher on Day 3 (12.5%—30 larvae) than on Day 2 (2.5%—7 larvae). Although significantly fewer larvae resumed swimming than were rehydrated on both days (*P* < 0.05), there was no significant difference in recovery between larvae from Day 2 and larvae from Day 3 (*P* > 0.05).

## Discussion

We have shown for the first time that coral larvae can survive low-temperature cryopreservation and resume swimming after warming. This achievement represents a crucial first step toward biobanking of larvae as a means of preserving the genetic diversity of coral, and provides important biological and physical information that will enable the development of high-throughput processing to support research and restoration efforts.

The results of the present study indicated that larval age affected the permeability of water and cryoprotectants across larval cell membranes, along with the ability of the larvae to withstand cryoprotectant solution toxicity and the stresses of vitrification and warming. These differences coincided with increasing larval complexity from Day 2 to Day 3 as mesenteries developed within the gastrovascular cavity and internal structures developed and became more defined (Fig. 1; Supplemental Information). As with previous studies^21,22^, the movement of water across larval membranes in response to cryoprotectant exposure was extremely rapid at all larval stages assessed, but the permeability of the cryoprotectants DMSO and PG was reduced with increasing larval age (Fig. 2). The difference in volume response of larvae to the complex cryoprotectant solution, which was greater on Day 2 (34%) than on Day 3 (22%) (Fig. 4A), may reflect differences in the amount of dehydration that was occurring at each larval stage. Reduced dehydration would increase the likelihood of internal ice crystal formation during cooling and warming, and could explain why larval post-warming recovery was higher on Day 2 (43%) compared to Day 3 (37%). Based on the rates of water and cryoprotectant movement observed in response to individual cryoprotectants (Fig. 2) we have assumed that the movement of water out of cells was essentially complete before significant cryoprotectant permeation occurred. However, the precise rates of water (L_P_) and cryoprotectant (P_S_) permeability at each larval stage are yet to be determined, so it is possible that there was some degree of concurrent water and solute exchange.

Minimal re-swelling was observed in larvae from Day 2 and Day 3 that were exposed to complex cryoprotectant solution (Fig. 4A), which suggests that there was very little cryoprotectant permeation into larval tissues prior to vitrification. Permeation of cryoprotectants across cell membranes can occur via passive diffusion or via trans-membrane channels such as aquaporins (AQP). While some aquaporins are specific to water (e.g. AQP1 and AQP2), others (e.g. AQP3 and AQP7) allow the passage of small molecules such as glycerol and urea^23,24^. In mammalian embryos, PG moves across cell membranes primarily via passive diffusion, while DMSO is thought to use a combination of passive diffusion and facilitated transport via non-aquaporin channels^25^, and has also been shown to permeate via AQP3 expressed in *Xenopus* embryos^26^. Increasing solute concentration, in particular DMSO, is known to reduce the movement of water and solutes across the plasma membrane via a mechanism referred to as ‘osmotic clamp’^27^. The higher solute concentration in the complex cryoprotectant solution compared to the individual cryoprotectant solutions may have caused a similar membrane response in the coral larvae, which could account for the observed lack of cryoprotectant permeation in the complex solution. The high rate of water movement observed in all larval stages tested suggests the possibility of facilitated transport across the membranes, though the presence of aquaporins or other trans-membrane channels has not been confirmed in coral.

The main requirement of successful low-temperature cryopreservation is to prevent the formation of intracellular ice crystals that can damage and kill cells^28^. Vitrification avoids ice crystal formation by using high cryoprotectant concentrations and rapid cooling rates to transition directly from a solution to a clear, non-crystalline, solid state resembling glass^29^. As in the present study, cryoprotectant concentration can be reduced to minimize toxicity to cells and still achieve vitrification, but this increases the formation of ice nuclei during cooling and necessitates rapid warming rates to prevent the growth of ice crystals^30^. Infrared laser warming can warm samples at up to 10,000,000 °C/min^20^, depending on the energy applied to the sample and the amount of laser absorbing particles present. The concentration of gold nanoparticles and laser settings used in the present study were selected to produce the minimal amount of warming required to consistently melt the 1-µL sample droplet to avoid overheating and causing damage to the larvae. While these settings were sufficient to prevent devitrification during warming in Day 2 larvae, they may not have been sufficient to completely prevent the recrystallization of ice nuclei resulting from the reduced level of dehydration in Day 3 larvae. It is possible that further optimization of vitrification solution composition and laser parameters may improve the survival of larvae, particularly in later developmental stages.

The fact that larvae were able to resume swimming indicates that they survived vitrification and laser warming, but the best indicators of long-term competence are the ability of the larvae to take on their algal symbionts (family Symbiodiniaceae) and to settle. Unfortunately it was not possible to assess these parameters in a meaningful way during the present study due to the relatively small number of laser-warmed larvae generated. Larval settlement and metamorphosis to form a polyp are critical stages of development in reef-building corals, but only a small proportion of the larvae produced during spawning will go on to become ‘recruits’ on the reef. The application of larval cryopreservation to support research and reef restoration will therefore require the ability to freeze and warm hundreds of thousands of larvae at a time to account for this expected attrition. Toward this end, we have begun work on methods for high-throughput processing that will enable cryopreservation of large numbers of larvae from the same developmental stage. Additional challenges that will need to be addressed for future biobanking efforts include the presence of Symbiodiniaceae in larvae from species that have vertical transmission of algal symbionts (which may affect cryopreservation) and the variation in larval sizes among coral species. The *F. scutaria* larvae used in the present study were relatively small in diameter (~100–200 µm) compared to acroporid species (~400–600 µm), which are the predominant reef building coral on many reefs worldwide and are therefore an important target for biobanking. Cryopreservation of these larvae may require the development of new cryoprotectant solutions and warming parameters, and preliminary work on this has begun.

The ultimate survival of coral species and the ecosystems that they support will depend on long-term international efforts to minimize the effects of climate change. In the meantime, steps must be taken to preserve the biodiversity of coral reef ecosystems while broad genetic diversity still exists, so that future efforts to recover and restore damaged reefs will have access to as much diversity as possible. There are challenges that will need to be met before cryopreservation of coral larvae can be applied at the scale required for reef restoration, but with continued development, this exciting technique could become an invaluable research and conservation tool to help restore and diversify wild reef habitats.

## Materials and Methods

### Animal husbandry

To maximize genetic diversity, adult mushroom coral, *Fungia scutaria*, were collected from six different shallow patch reefs in Kaneohe Bay, Hawaii, and then maintained in an open seawater system at the Hawaii Institute of Marine Biology on Coconut Island (21° 26′ N, 157° 47′ W). A two- to three-night spawning period commenced two nights after the full moon in the months of June to September at approximately 17:00 h. On nights of anticipated spawning, individual corals were isolated in 2-L glass bowls containing 0.5-µm-filtered seawater. Although animal care and use protocols are not required for invertebrates, all efforts were made to ensure the ongoing health and viability of the animals.

### Fertilization

On release, sperm were aspirated from the polyp mouth with a transfer pipette and collected into a 50-mL tube, while eggs were allowed to settle at the bottom of the bowl and were collected after spawning was complete. Within 30 min, sperm motility from individual males was assessed at ×200 magnification with an Olympus BX41 compound microscope (Center Valley, PA, USA) by estimating the average of the proportion of moving sperm in at least 3 to 5 fields. Samples with sperm motility ≥50% were pooled (4–6 males per spawning day). Thousands of eggs from each individual female (5–7 females per spawning day) were gently transferred to 3-L plastic bowls containing 0.5-µm-filtered seawater and fertilized by adding pooled sperm at a final concentration of approximately 5 × 10^5^ to 1 × 10^6^ cells/mL. After 1 h the mixture was rinsed by removing approximately 75% of the seawater and sperm from the bowl and replacing it with clean 0.5-µm-filtered seawater. Bowls containing thousands of fertilized eggs each remained on a shaded bench-top at ambient temperature (24–26 °C) with no water flow or aeration. Water changes were performed daily by collecting larvae into a 10-cm diameter, 40-µm mesh filter basket, rinsing them with FSW, and transferring them into a clean bowl with fresh FSW. Larvae produced from a total of 16 males and 24 females that spawned during July and August 2017 were used for shrink/swell, cryoprotectant toxicity, and laser warming experiments.

### Media preparation

Dimethyl sulfoxide, propylene glycol, and phosphate buffered saline were obtained from Sigma-Aldrich (St. Louis, MO, USA), and trehalose was from Pfanstiehl Inc. (Waukegan, IL, USA). Gold nanorod particles were obtained from nanoComposix (San Diego, CA, USA). Seawater used in spawning bowls and for larval rearing was filtered with a canister filter system containing 20-µm and 0.5-µm filter cartridges. Seawater for larval experiments was filtered with a 0.22-µm nitrocellulose filter (Merck Millipore, Billerica, MA, USA) and used on the day of preparation.

### Actin staining

Actin in the developing larvae was labelled using Alexa Fluor 488-conjugated phalloidin (Molecular Probes, Eugene, OR, USA) to define the development of the gastrovascular cavity, as described by Marlow *et al*.^31^, and imaged using an Olympus BX51 microscope fitted with an Olympus MagnaFire SP camera and imaging software.

### Larval volume response to individual cryoprotectants

We measured volume changes over time in two- to five-day-old *F. scutaria* larvae exposed to individual permeating (DMSO and PG) and non-permeating (trehalose) cryoprotectants. Each cryoprotectant solution was prepared at double the treatment concentrations (20% v/v DMSO in FSW, 20% v/v PG in FSW, and 1.5 M trehalose in deionized water). For treatment, larvae (n = 5–10) from a pool of 4–5 females were transferred in 5 µL FSW onto a well slide. An equal volume of cryoprotectant solution was added to the well and mixed by gentle pipetting to create final treatment concentrations of 10% v/v DMSO in FSW, 10% v/v PG in FSW, and 0.75 M trehalose in 50% FSW. The moment that the mixing began was recorded as t = 0. A cover slip was placed over the well once mixing was complete and images were captured in dark field at ×100 magnification on a compound microscope using a Lumenera Infinity 3s camera (Ottawa, ON, Canada) connected to Infinity Analyze capture software. Images were taken at 5-second intervals for a total of 180 seconds. The time taken to place a coverslip and allow the sample to stabilise meant that the first useable image was collected at 10 seconds after cryoprotectant addition. Larvae that overlapped or touched the edge of the microscope field during treatment were excluded from analysis, so it was usually only possible to measure volume changes in 1–5 larvae per well. Exposures were repeated for each larval stage until a total of 10–15 larvae were imaged for each cryoprotectant solution. For the control, a total of 10–15 larvae were exposed to FSW alone and a single image was recorded since volume was not expected to change over time.

Image sequences of larvae in each cryoprotectant solution and in FSW were analyzed in ImageJ software (National Institutes of Health, USA). Each image was reduced to 8-bit grayscale and individual larvae within each image were identified by x/y coordinates and tracked over time. The semi-minor (a) and semi-major (c) axes, in linear pixels, of the closest-fit ellipse to the cross-section of each larva were returned by ImageJ. Linear pixels were converted to µm based on a calibration image of a slide micrometer, and larval volumes at each time point were calculated based on modeling each larva as a prolate spheroid (V = (4π/3)a^2^c). Analysis of most treatments began at t = 10 s as the time required to mix the sample, apply the coverslip, and bring the cells into focus usually precluded capture of earlier data. As it was not practical to take images of the same larvae before and after treatment, the t = 0 volume for treated larvae was assumed to be the mean volume of the FSW control. The response of larvae to the cryoprotectants was tracked over time by plotting changes in larval volume. This information, along with observations of physical changes to larval membranes, was used to determine exposure times to cryopreservation solutions in subsequent toxicity and vitrification experiments.

### Cryoprotectant toxicity

Based on the response of larvae to individual cryoprotectant solutions, two complex cryoprotectant solutions capable of vitrification were designed. Solution 1 consisted of 7.5% v/v DMSO, 7.5% v/v PG, and 1 M trehalose in PBS, and solution 2 consisted of 5% v/v DMSO, 10% v/v PG, and 1 M trehalose in PBS. Both solutions contained a total of approximately 3.5 M solutes. The ability of these solutions to vitrify was confirmed by placing a 1-µL droplet of cryoprotectant solution onto the blade of a cryotop (see description below) and plunging it directly into liquid nitrogen. The solution was considered to be vitrified if the droplet remained clear with no signs of haziness or cracking after immersion in liquid nitrogen with visual inspection.

Cryoprotectant toxicity experiments were conducted when larvae were around 36 hours post-fertilization (considered ‘Day 2’ larvae) and repeated at approximately 24-hour intervals for days 3, 4, and 5. Larvae (20–40 per replicate, 3 technical replicates per larval stage) pooled from 10 females were transferred into a 40-µm cell strainer in FSW. The cell strainer was blotted to remove excess FSW then transferred into cryoprotectant solution 1 or 2 in the first well of a 6-well plate for 60 seconds. Larvae were rehydrated by transferring the filter basket through a series of wells containing 75, 50, and 25% cryoprotectant solution diluted in FSW for one minute each, ensuring that the filter basket was blotted between wells. After the final rehydration solution, the cell strainer with larvae was blotted again, rinsed briefly by dipping in FSW to remove any remaining cryoprotectant solution, and transferred to a clean 6-well plate with FSW. The 6-well plate was kept in an incubator at 26 °C to allow larvae to recover. Larvae were observed with an Olympus SZX12 dissecting microscope at ×50 magnification and survival was assessed by counting the numbers of moving and non-moving larvae at 1 hour and 2 hours post-treatment.

### Vitrification and laser warming

Vitrification and laser warming were performed essentially as described by Khosla *et al*.^18^, with some modifications. Vitrified samples were warmed using a bench-top iWeld 980 Series, 60 joule, Nd:YAG infrared laser (LaserStar Technologies Corporation, Orlando, FL, USA). The laser is equipped with a stereomicroscope with an eyepiece crosshair reticule to allow visualization and alignment of samples within the laser chamber. A custom-made cryo laser apparatus (Design Solutions, Inc., Chanhassen, MN, USA) was used to lower the sample into liquid nitrogen for vitrification, raise the sample into the laser beam focal region, and trigger the laser for warming. The apparatus was inspired by the Cryojig^©^ described by Kleinhans and Mazur^32^, and consisted of a digital servo (HS-5625MG, Hitec RCD USA Ltd., Poway, CA, USA) fitted with a 3D-printed plastic arm designed to hold a cryotop. The servo motor was attached to an x/y stage with a 3D-printed plastic base and the whole apparatus was positioned above a 10 (w) × 15 (l) × 5 (h) cm polystyrene bath containing liquid nitrogen. A control box with custom software (Design Solutions, Inc., Chanhassen, MN, USA) was used to control the position of the arm holding the cryotop and to fire the laser. For warming of vitrified larvae the control box was used in ‘AUTO’ mode so that a single laser pulse was fired automatically when the sample was raised into the laser beam focal region.

Gold nanorods, as characterized by Khosla *et al*.^18^, were used as a laser absorber for warming of vitrified samples. The nanorods were diluted with cryoprotectant solution at a ratio of 1:29, for a final concentration of approximately 1.75 × 10^11^/mL (0.0625 mg/mL). This cryoprotectant solution containing gold nanorods was then used for vitrification and laser warming of *F. scutaria* larvae. A laser calibration table was generated using a laser power meter (Nova II, Ophir, Jerusalem, Israel) to determine the amount of energy in joules produced by the iWeld 980 Series laser at a given voltage and pulse time. The laser energy range required to melt a 1-µL vitrified droplet of cryoprotectant solution was calculated based on the absorption properties of the gold nanorods and the laser energy output. Final settings (360 V, 2.5 ms pulse time, and 2 mm laser spot diameter) were determined empirically by vitrifying 1 µL droplets of cryoprotectant solution on cryotops and observing the outcome when the droplet was warmed by the laser. The cryotops used for vitrification were a modified version of the Kitazato Vitrification Cryotop^®^ (Kitazato, Tokyo, Japan) that have been used previously for successful vitrification and laser warming of oocytes and embryos^18,19,32^, and consisted of a 1.5 × 10 mm acetate blade mounted in a wooden press-fit holder. This system was developed after it was discovered that the laser pulse caused pitting and melting of the cryotop blade requiring regular replacement of the cryotop to avoid variation in sample warming. The modified version allowed easy replacement of the cryotop blade after each laser pulse to ensure consistent and reproducible sample warming. Distribution of the gold nanorod particles around the larvae was assessed using gold nanorods coated with DyLight 650 (nanoComposix, San Diego, CA, USA) diluted with cryoprotectant solution at a ratio of 1:29. A 1-µL droplet of cryoprotectant containing the DyLight-coated gold nanorod particles and several *F. scutaria* larvae was imaged using a Zeiss LSM 710 confocal microscope. Green fluorescent protein in the larvae was excited with a 405 nm laser and detected with a 410–626 nm emission filter setting, and the DyLight-coated gold nanorods were excited with a 633 nm laser and detected in the 641–729 nm range.

A random selection of pooled Day 2 or Day 3 larvae was concentrated in a 40-µm cell strainer in a 35-mm dish with FSW. Handling of larvae for vitrification and rehydration was performed under a dissecting microscope at ×40 magnification. A pipette was used to collect up to 20 larvae in 1 µL of FSW and transferred to the lid of a 35-mm dish. The volume of FSW was carefully reduced to approximately 0.5 µL using a pipette and a 5-µL droplet of cryoprotectant solution containing gold nanorods was added to the larvae and gently swirled with the pipette tip to ensure mixing. The larvae were collected into 1 µL of cryoprotectant solution and placed on the flat blade of a modified cryotop, which was loaded into the cryo laser apparatus. The droplet was checked quickly through the iWeld stereomicroscope to confirm the number of larvae (mean ± S.E.M. = 12.5 ± 0.5 larvae, range 8–20 per droplet) and to ensure that the sample was positioned in the laser crosshairs, then lowered into the liquid nitrogen and vitrified. After 1 minute in liquid nitrogen, the sample was raised and warmed with a single infrared laser pulse of 10.9 J of energy, corresponding to a warming rate of approximately 4,500,000 °C/min. The sample was viewed through the iWeld stereomicroscope during warming and samples that melted completely with no signs of devitrification or refreezing were removed from the laser chamber for rehydration of the larvae. For rehydration, larvae were transferred through a series of 100-µL droplets, consisting of 75, 50, and 25% (v/v) cryoprotectant solution diluted with FSW, for one minute each. In each droplet the larvae were swirled gently then collected in 1 µL and moved to the next solution. After the 25% cryoprotectant solution, larvae were collected in 1 µL and transferred to 2 mL of FSW in a well of a 24-well plate. Larvae were allowed to recover overnight in an incubator at 26 °C, and survival was assessed at 12–18 hours after warming as described.

### Statistics

Prior to statistical analyses, percentage data from the cryoprotectant solution toxicity experiment were transformed to arcsine values, and count data from the vitrification and laser warming experiment were transformed to square-root values. Gaussian distribution was assessed using the Shapiro-Wilk normality test. Both data sets were analyzed by Kruskal-Wallis test followed by Dunn’s multiple-comparisons test using GraphPad Prism version 7 for Mac OS X. Differences were considered significant at *P* < 0.05, and all data are presented as mean ± SEM.

## Electronic supplementary material

SUPPLEMENTAL INFORMATION
