# Supplementary material for: Successful cryopreservation of coral larvae using vitrification and laser warming
Source: Sci Rep. 2018 Oct 24;8:15714. doi: 10.1038/s41598-018-34035-0 (PMC6200823; doi:10.1038/s41598-018-34035-0)
Supplement: Supplementary file 1 — SUPPLEMENTAL INFORMATION [file 41598_2018_34035_MOESM1_ESM.docx]

**** SUPPLEMENTAL INFORMATION ****

**Successful cryopreservation of coral larvae using vitrification and laser warming**

Jonathan Daly^1,2^, Nikolas Zuchowicz^1,2^, Carmela Isabel Nuñez Lendo^1,2^, Kanav Khosla^3^, Claire Lager^1,2^, E. Michael Henley^1,2^, John Bischof^3,4^, Frederick W. Kleinhans^5^, Chiahsin Lin^6,7^, Esther C. Peters^8^, and Mary Hagedorn^1,2^

^1^ Smithsonian Conservation Biology Institute, Front Royal, VA 22630, United States of America

^2^ Hawaii Institute of Marine Biology, 46-007 Lilipuna Rd, Kaneohe, HI 96744, United States of America

^3^ Department of Mechanical Engineering, University of Minnesota at Twin Cities, 111 Church St SE, Minneapolis, MN 55455, United States of America

^4^ Department of Biomedical Engineering, University of Minnesota at Twin Cities, 312 Church St SE, Minneapolis, MN 55455, United States of America

^5^ Department of Physics, Indiana University-Purdue University Indianapolis, Indianapolis, IN 46202, United States of America

^6^ National Museum of Marine Biology & Aquarium, Pingtung 944, Taiwan

^7^ Institute of Marine Biology, National Dong Hwa University, Pingtung 944, Taiwan

^8^ Environmental Science and Policy, George Mason University, Fairfax, VA 22010, United States of America

**Corresponding author:**

Jonathan Daly, Smithsonian Conservation Biology Institute, Front Royal, VA 22630, United States of America. TEL: (808) 825-1596 EMAIL: dalyj@si.edu

**Histology materials and methods**

Larvae at various developmental stages were processed for histology and antibody staining. To embed in paraffin, larvae were collected into 40-µm cell strainer baskets (Falcon, Fisher Scientific) and transferred into 2% glutaraldehyde in FSW for 12 h at 4°C. The fixed larvae were rinsed 3–4 times with FSW and stored at 4°C until processing (less than 2 weeks). For antibody processing, larvae were collected in cell strainer baskets in FSW then transferred into 4% paraformaldehyde (EMS, Hatfield, PA) for 45 min at RT. They were then rinsed 3–4 times in PBS (1 min each) and stored in fresh PBS at 4°C.

Fixed larvae were enclosed in an envelope folded from lens paper (ca. 4 cm x 6 cm) to prevent loss and damage during processing and placed into a microcassette. The lens paper did not inhibit the infiltration of solutions into the larvae; however, processing time was increased to allow for complete diffusion of solutions through the paper. The microcassettes were placed into 70% ethanol overnight, then dehydrated through a series of alcohols (3 × 40 min for 95% ethanol, 3× 40 min for 100% ethanol), followed by xylene (3 × 40 min), and finally infiltrated with molten (60°C). Paraplast (3 × 40 min). The larvae were stained with 1% alcoholic eosin Y (Fisher Scientific) for 1 min between the final 95% and first 100% ethanol solutions to increase visibility in the paraffin blocks.

To transfer the larvae from the lens paper envelopes into the final paraffin blocks, the envelopes were opened using warm forceps and inverted into a metal dish containing warm paraffin. Collected larvae and paraffin were poured into warm stainless-steel molds, ensuring that all the larvae had sunk to the bottom of the mold (i.e., the face of the block), then allowed to set. Sections were cut at 6 µm using a rotary microtome, collected onto untreated clean glass slides, and dried overnight on a slide warming tray at 45°C. Sections were stained with Harris’s hematoxylin, then dehydrated and cover-slipped with Permount Mounting Medium (Fisher Scientific, Hampton, NH, USA). Tissue was examined with a compound microscope at ×1000 magnification under oil-immersion and images were taken using an Olympus camera. Sections were examined for the development of endoderm, ectoderm, and mesoglea, and the appearance of the mesenteries and adult differentiated cell types in the larval epithelia, such as mucocytes, neurons, epitheliomuscular cells, ciliated cells, and cnidocytes.

**Histology results**

The first 24 h is a period of rapid cellular development and differentiation in the larval *F. scutaria* (Fig. 1 A, B). At 24 h, the cells are filled with yolk granules to feed the developing larva. A ciliated actinopharynx is beginning to form, and the ectoderm, endoderm, and the mesoglea can be distinguished. The nuclei in these epithelia are pale with prominent nucleoli. Rounder nucleated cells that can migrate between the epithelial cells, called interstitial (stem) cells, may possibly be forming the epithelial cells. All cellular division is not complete at this time, because mitotic figures were observed in the apical portion of the epithelia in the sections. Similarly, differentiation is not complete because there are undifferentiated columnar ectodermal cells and endodermal cells. The invagination for a mouth is evident and the apical cilia of the endodermal cells intrude into the lumen that will develop into the gastrovascular cavity. In some sections, large cells positioned in the ectoderm appeared to be developing mucocytes. When stressed the larvae can form mucus-like substances, so some cells may be functional, but not abundant at this time.

At Day 2, more prominent linkages have formed between the cells in the ectoderm, with more columnar cells and interstitial cells present. Neuroblasts were found in the ectoderm and endoderm, and the first nematoblasts were seen. The apical surfaces of the ectodermal columnar cells have become more pronounced as a brush border. Mucocytes are present and appear relatively well-differentiated. The endoderm continues to develop and a lumen for the gastrovascular cavity is evident (Fig. 1 C, D)

On Day 3, the endodermal cells have a more mature morphology and clear vesicles likely filled with lipids were found in the basal portions of those cells. The external epithelial cells are elongated and compacted, (Fig. 1 E, F), and cnidogladular bands and mesenteries are beginning to form. The organelles of nematocytes, specifically holotrichous isorhizas, appeared.

On Day 4, much of the yolk proteins have been absorbed by the endodermal cells, the ectoderm seems to be even more sharply defined with numerous differentiated cells present, the mesoglea has thickened and developed more fully, and continuing mesentery formation was observed (Fig. 1 J, K).


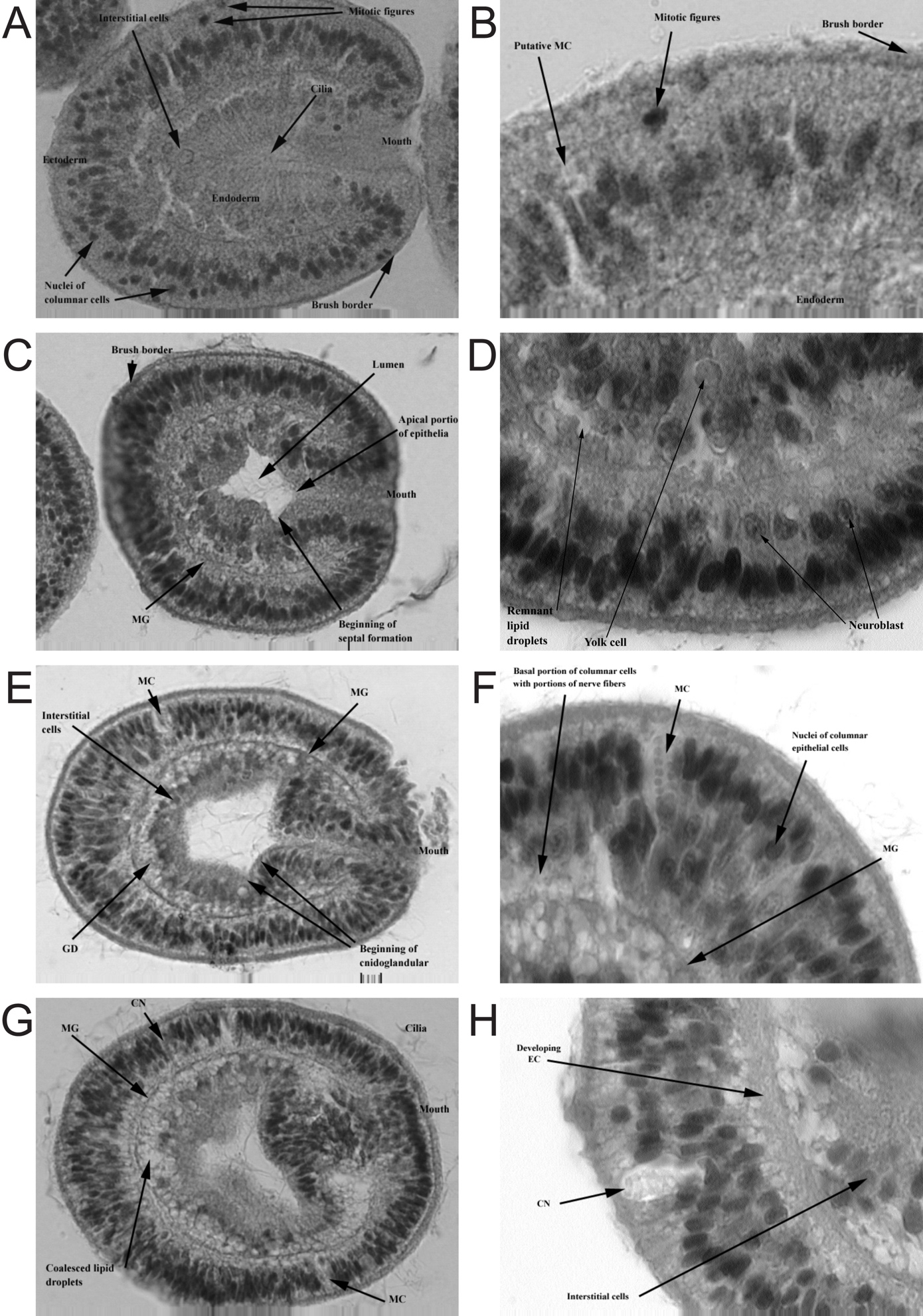


**Figure 1:** Developmental sequence of *F. scutaria* from Day 1 to 4. Lower power longitudinal images are in the left column at the same magnification (bar = 40 µm). The apical region is to the right and the basal to the left. Enlargements of each developmental stage are on the right, all at the same magnification (bar = 20 µm). **A.** Day 1, the ectoderm is differentiated from the endoderm and the mesoglea has begun to form. There is no opening of the actinopharynx. The nuclei of the epithelial columnar cells are seen, as well as mitotic figures of dividing cells. The brush border appears as a darkened line, and the external cilia as thin fibers from that border. **B.** Day 1 larvae were observed producing mucus and putative mucocytes can be seen in the ectoderm. **C.** Day 2, the mesoglea becomes more clearly defined. The gastrovascular cavity begins to open and the mesenteries begin to form. **D.** Day 2, as the larvae develop the lipid droplets start to coalesce. Remnant yolk cells are seen, as well as the developing neuroblasts. **E.** Day 3, the actinopharynx begins to widen and open. The cnidoglandular band forms along the free edges of mesenteries as they develop. The ectoderm becomes more complex and pseudostratified. The mesoglea appears as a well-defined darkened band. **F.** Day 3, pseudostratified columnar epithelium of ectoderm is more easily visible. Basal nerve net begins to form. **G.** Day 4, as well as mucocytes, cnidoblasts can be seen in the ectoderm. **H.** Day 4, the developing muscular layer (myonemes) can be seen along the base of the columnar epitheliomuscular cells adjacent to the mesoglea.

KEY: CN - cnidoblasts

EC - epitheliomuscular cells

GD - gastrodermis

MG - mesoglea

MC - mucocytes
